# Association of Schimmelpenning Syndrome with Astrocytoma (WHO Grade 3): Case Report

**DOI:** 10.3390/medicina60101688

**Published:** 2024-10-14

**Authors:** Aija Tumova, Kaspars Auslands, Andrejs Millers, Zanda Priede, Māris Buks, Agnese Ozola, Elīna Ozoliņa, Kārlis Bicāns, Rūdolfs Ulmanis

**Affiliations:** 1 Department of Neurology and Neurosurgery, Riga Stradins University, 1007 Riga, Latvia; kaspars.auslands@rsu.lv (K.A.); ulmanis90@gmail.com (R.U.); 2 Department of Neurosurgery, Riga East Clinical University Hospital, 1038 Riga, Latvia; 3 Department of Neurosurgery, Pauls Stradins Clinical University Hospital, 1002 Riga, Latvia

**Keywords:** Schimmelpenning syndrome, astrocytoma, nevus, HRAS gene

## Abstract

Schimmelpenning syndrome, or epidermal nevus syndrome, is a rare, neurocutaneous disorder characterized by skin abnormalities, such as epidermal nevi, and involvement of the central nervous system, including intracranial tumors. There are only a few reported cases of intracranial tumors associated with Schimmelpenning syndrome. In most cases, a single nucleotide mutation in the RAS family proto-oncogenes, like *HRAS* or *KRAS* genes, can result in the genetic mosaicism that is responsible for the clinical manifestations of this syndrome. The authors present a case report of a woman with Schimmelpenning syndrome who sought medical help with complaints of progressive headache and dizziness. The radiological and histopathological findings indicated an astrocytoma, IDH-mutant (WHO grade 3). The molecular analysis revealed pathogenic changes in the oncogenic *HRAS* gene with a prevalence of 31%. The patient underwent surgical treatment and had no neurological sequelae. By presenting such a clinical case, attention is paid to the interrelationship between genetic syndromes and intracranial tumors.

## 1. Introduction

Schimmelpenning syndrome, also known as Schimmelpenning–Feuerstein–Mims syndrome or epidermal nevus syndrome, is a rare, congenital disorder that is considered a neurocutaneous pathology and characterized by the presence of skin abnormalities, ocular and skeletal defects, and involvement of the central nervous system [1,2].

The main features of Schimmelpenning syndrome include the following: 

(1) Epidermal nevus, characterized by thickened, hyperpigmented skin patches which can vary in size and distribution;

(2) Central nervous system (CNS) involvement, which may include brain malformations, seizures, developmental delay, and intellectual disabilities. While not every individual with Schimmelpenning syndrome will develop intracranial tumors, the syndrome can present with tumors, which contribute to its neurological features.

(3) Additional features may include skeletal, cardiovascular, and ocular anomalies. The specific manifestations can vary among patients [3,4].

The syndrome is thought to be caused by mutations in the *HRAS* gene. *HRAS* activation has been reported in several tumor types and can lead to the activation of signaling pathways, which play a role in cell growth and division [5]. In Schimmelpenning syndrome, an activating mutation in the *HRAS* gene is thought to contribute to the development of epidermal nevi and other features characteristic of the syndrome [3,5].

Astrocytomas, on the other hand, are tumors that arise from astrocytes, a subtype of glial cells in the human CNS. They are classified by the World Health Organization (WHO) into different grades based on their molecular and genetic characteristics, using a combination of histology and molecular markers, with grade 3 astrocytomas being considered malignant [2,5,6]. It is important to note that the molecular mechanisms underlying the relationship between genetic mutations, such as *HRAS* mutations, and the development of tumors, including astrocytomas, can be complex and may involve multiple factors [7]. This article describes a rare case of a 44-year-old woman who is diagnosed with Schimmelpenning syndrome and an intracranial tumor (astrocytoma, grade 3).

## 2. Case Presentation

**Figure 1:**
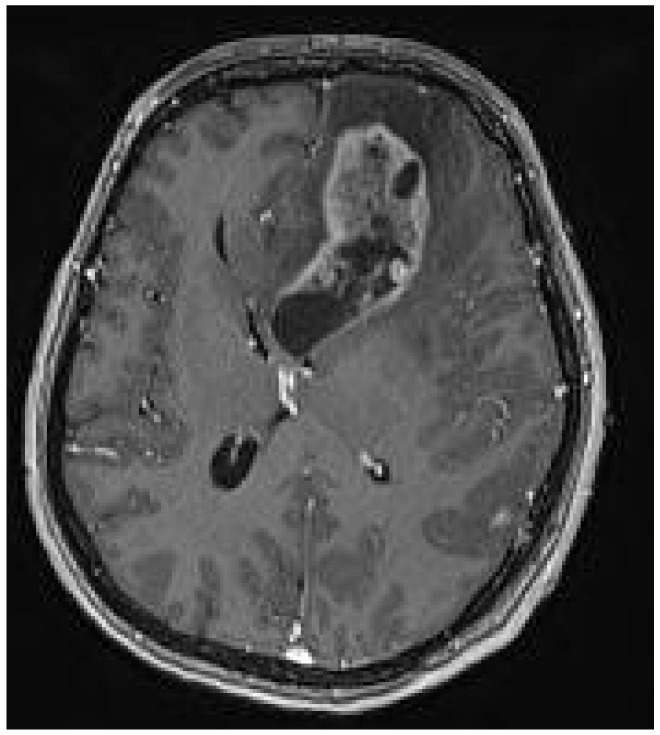
Preoperative MRI T1 with contrast. Axial plane. A 6.5 × 3 × 3 cm irregular-sized, nonhomogeneous pathologic mass in the left frontal lobe with a white matter interstitial edema around it. The pathologic mass deforms and partially compresses the left lateral ventricle, with dislocation to the right side (19 mm). Dislocation of the septum pellucidum (12 mm) and the third ventricle (5 mm) to the right side.

A 44-year-old female patient with previously diagnosed Schimmelpenning syndrome sought emergency medical help with complaints of progressive headache and dizziness. Magnetic resonance imaging (MRI) of the brain revealed a 6.5 × 3 × 3 cm irregularly shaped, inhomogeneous pathological mass with a cystic component that contrasted inhomogeneously in the left frontal lobe (Figure 1). The mass effect of the growing tumor caused brain deformations, including left lateral ventricle partial compression, dislocation of the third ventricle to the right by 5 mm and a midline shift to the left, and septum pellucidum displacement to the right by 12 mm. Interstitial swelling of the white matter of the brain around the tumor was detected.

The patient underwent surgical treatment in May 2023. Osteoplastic trepanation was performed on the patient’s right frontotemporal bone. The dura mater was separated from the bone and cut open, exposing the brain. Tumor tissue was dissected under microscope control. It was possible to evacuate practically the entire tumor within the limits of visibility. The molecular analysis revealed pathogenic changes in the oncogenic *HRAS* c37G>C p.(G13R) with a prevalence of 31%. The p. G13R allelic variant results in a constantly active H-Ras protein, promoting uncontrolled cell growth and division, which can be associated with a more aggressive tumor type. Genetic changes in the *KRAS*, *NRAS*, and *BRAF* genes were not found. The histopathological analysis confirmed the sampled tissue to be an astrocytoma IDH mutant, grade 3.

**Figure 2:**
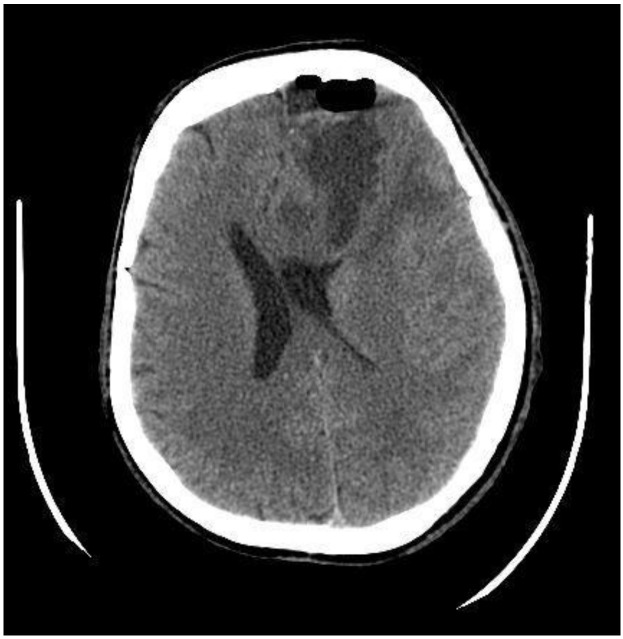
Postoperative CT scan, axial plane, after tumor resection from the left frontal lobe with frontal bone osteoplastic trepanation. Cystic tumor residual tissue, edema, and hemorrhage can be seen in the surgical site. The dislocation of the left lateral ventricle has decreased to 14 mm (preoperatively 19 mm).

A postoperative CT scan of the brain showed decreased midline dislocation and minimal blood content in the remnant tumor tissue (Figure 2). 

**Figure 3:**
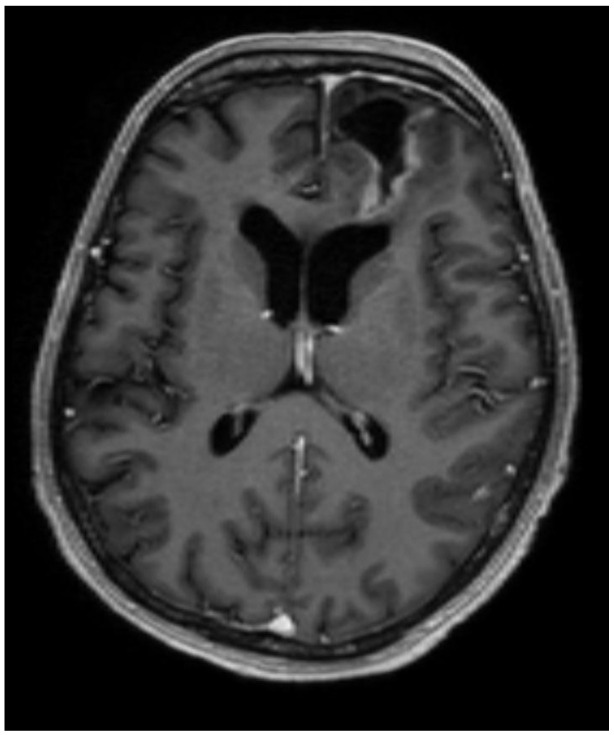
The postoperative MRI, T1, axial plane, shows cerebrospinal fluid collection in the surgical site (31 × 35 × 19 mm) with encephalomalacia, hemosiderin sediments, and gliosis surrounding it, which reaches the lateral ventricle, gyrus rectus, and medial orbital gyrus. The midline and the left lateral ventricle are no longer dislocated. Residual tissue can be seen in the surgical site’s posterior, lateral, and medial walls (~10 mm dense).

After surgery, the patient’s complaints about headaches and dizziness decreased, and the patient had no neurological deficit. The patient was discharged from the hospital on the 14th postoperative day. A control MRI of the brain was performed in August 2023, which showed no data on the progression of the process, but there was residual tissue of the tumor (Figure 3). 

**Figure 4:**
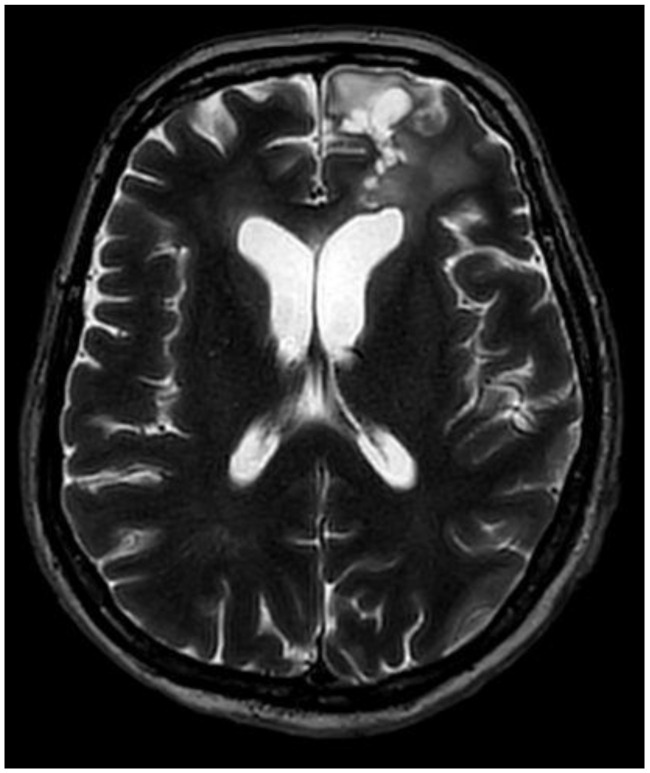
Postoperative MRI, T2, axial plane, after nine months. The size of the surgical site cavity has decreased to 29 × 24 × 15 mm. Perifocal gliosis and an encephalomalacia zone can still be detected in the left lateral ventricle, gyrus rectus, and medial orbital gyrus. The residual tissue has decreased in size and can only be seen in the posterior wall of the surgical site cavity.

**Figure 5:**
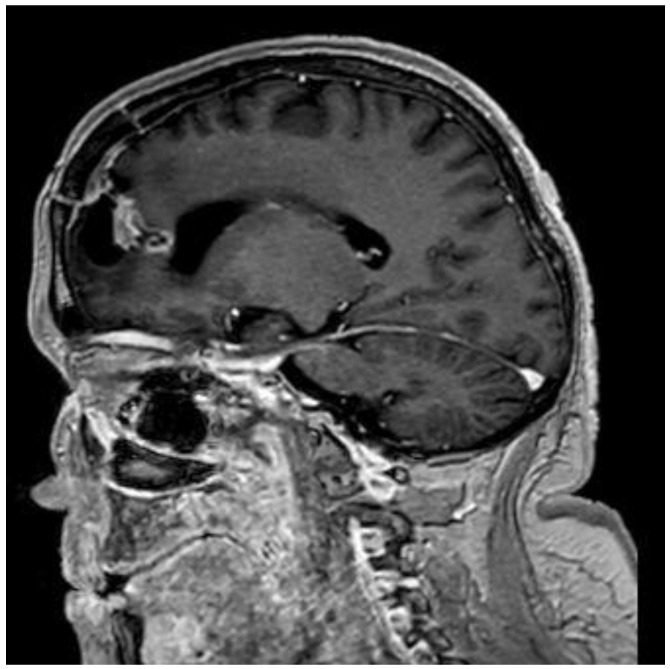
Postoperative MRI, T1, sagittal plane, after 9 months. The residual tissue has decreased in size and can only be seen in the posterior wall of the surgical site cavity.

A multidisciplinary neuro-oncology council reviewed the patient’s case, agreeing on further therapy. The patient received combined radiation and chemotherapy, which she tolerated well; the total radiation dose was 40.05 Gy, and Temozolomide was administered at 100 mg per day for 25 days. The patient was discharged from the hospital in good condition with no neurological or cognitive deficits, with recommendations for further observation under the supervision of a general practitioner. A control MRI scan was performed 9 months after the surgery, and no evidence of tumor progression was noted (Figure 4 and Figure 5).

## 3. Discussion

This case describes a patient with a clinical diagnosis of Schimmelpenning syndrome who presented with progressive headache and dizziness secondary to an IDH-mutant astrocytoma, grade 3. The patient did not have a neurological deficit upon admission.

Data from literature sources indicate that the majority of previously reported cases of Schimmelpenning syndrome have neurological features, including seizures, intellectual disability, hemiparesis, lateral ventricle enlargement, and complete or partial hemimegalencephaly [8]. Tissue samples from lesions of patients with Schimmelpenning syndrome have mutations in either the *HRAS* or *KRAS* gene [2]. In the 2012 report, which included 65 patients with sebaceous, 95% (*n* = 62) had mutations in the *HRAS* gene, but only 5% (*n* = 3) had mutations in the *KRAS* gene. The main mutation in *HRAS,* c.37G>C, causes a p.G13R substitution that constitutively activates the Ras/Raf/MAPK signaling pathway [7]. This mutation was present in 93% of lesions and was associated with secondary tumors in all cases, showing the etiology of Schimmelpenning syndrome developing as a result of genetic mosaicism. Therefore, it is an autosomal dominant RASopathy [8].

In this case of an adult woman with Schimmelpenning syndrome with an IDH-mutant astrocytoma, WHO grade 3, a mutation in the *HRAS* gene with 31% prevalence was found. No mutations in the KRAS, NRAS, and BRAF genes were found. 

**Figure 6:**
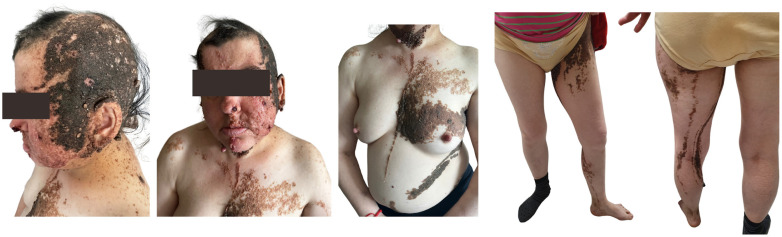
Patient (44 y.o. female) with Schimmelpenning syndrome. Diffusely located, multiple, irregularly shaped and sized, multicolored epidermal nevi with left-side predominance.

Schimmelpenning syndrome is associated with a wide range of abnormalities, most commonly presenting with skeletal, neurological, ocular, and extracutaneous features [9]. Our patient had skin abnormalities from birth, including large, hairless, brown plaques on the scalp and face, along with multiple brown verrucous plaques and brownish-black macules on the left side of the body (Figure 6).

Patients with Schimmelpenning syndrome may have neurological symptoms, which can appear over a period of time. Therefore, there is suspicion of intracerebral neoplasm growth associated with the neurological symptom complex, together with seizures and an increase in intracranial pressure [2,7,10].

There are some studies which show a link between Schimmelpenning syndrome and intracranial tumors. A summary of Schimmelpenning syndrome (Table 1) and related disorders (Table 2) with intracranial lesions is documented below.

Extracutaneous neoplasms affecting the central nervous system have rarely been observed in patients with Schimmelpenning syndrome. In one case report, a patient presenting with Schimmelpenning syndrome was diagnosed with pilocytic astrocytoma [2]. A second case report included a Schimmelpenning syndrome patient with parenchymal brain cysts [11]. Another report included Schimmelpenning-like syndrome with anaplastic astrocytoma, a linear syringocystadenoma papilliferum, and ocular abnormalities [13]. There is only one case report that describes the association between Schimmelpenning syndrome and intracranial tumors and the nature of their coexistence, the occurrence of pilocytic astrocytoma, and the Ras signaling pathway, suggesting that there is a common pathway in the pathophysiology of both diseases [2].

However, the uncommon relation between extracutaneous neoplasms and the neurological symptom complex needs more thorough monitoring and genetic testing of the affected tissues [1]. Genetic testing should be performed on affected tissues, evaluating for mutations in genes affecting the Ras/Raf/MAPK signaling pathways [7]. Grade 2 and grade 3 astrocytomas have a similar frequency of chromosome 7 gains and TP53 and IDH1 mutations, as well as allelic loss on chromosomes 6, 9p, 11p, 19q, and 22q [2]. The CDKN2A, p14ARF, and CDKN2B tumor suppressor genes are important targets for genetic and/or epigenetic inactivation, with the inactivation of p14ARF serving as an alternative means to impair the p53 pathway in cases without TP53 mutations. In anaplastic astrocytoma, RAS mutations typically involve alterations in the *HRAS*, *KRAS*, or *NRAS* genes. These mutations can occur through various mechanisms, including point mutations, insertions, deletions, and gene amplifications. The specific genetic alterations resulting from RAS mutations in anaplastic astrocytoma can vary among individual tumors, but they often lead to constitutive activation of RAS signaling pathways, promoting cell proliferation and tumorigenesis [9,10]. 

MRI of the brain is the gold standard for diagnosing glial tumors, including astrocytomas, oligodendrogliomas, and glioblastomas. It allows for the visualization of a tumor’s location, size, and involvement with the surrounding brain tissue and helps plan for a potential surgical intervention. Advanced MRI techniques can also be used, such as magnetic resonance spectroscopy (MRS), perfusion MRI, and diffusion tensor imaging (DTI), which can offer additional information about the tumor’s metabolic profile, blood flow characteristics, and involved white matter tracts [37]. However, a definitive diagnosis of glial tumors must be based on a histopathological examination of the tumor tissue obtained during a biopsy or surgical resection. Recent studies have also highlighted the role of molecular and genetic testing in diagnosing and classifying glial tumors, with implications for targeted therapy. Identifying specific genetic mutations has become an integral part of the diagnostic process, for example of isocitrate dehydrogenase (IDH) mutations, 1p/19q encoding, and MGMT promoter methylation [10,38]. 

From a molecular standpoint, IDH mutations have been shown to affect cellular metabolism, cancer biology, and oncogenesis [38]. They are prevalent in malignancies and are the leading cause of the development of astrocytomas. Typically, the IDH enzymes convert isocitrate to alpha-ketoglutarate. In the case of astrocytomas, the mutated IDH reduces alpha-ketoglutarate to D2-hydroxyglutarate, an oncometabolite that accumulates in the targeted tissue [39]. IDH mutations are recognized in over 80% of WHO grade II or III cases [38].

**Figure 7:**
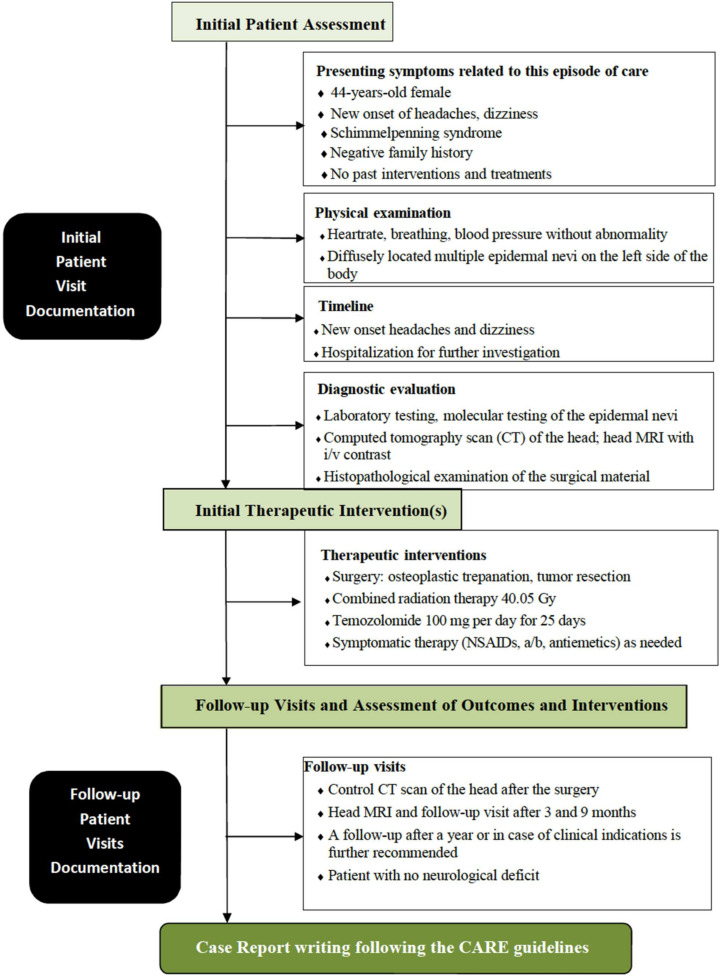
Flowchart outlining the diagnostic procedure steps in this case, which begin with a patient presenting with symptoms and involve developing an examination and treatment plan, including operative therapy, followed by specific treatment and further follow-up of the patient.

The surgical strategy for managing an intracranial astrocytoma, WHO grade 3, involves a multidisciplinary approach aimed at maximal safe tumor resection to facilitate further adjuvant therapies and improve patient outcomes [6,38,39]. Given the aggressive nature of grade 3 astrocytoma, surgery is typically followed by radiation therapy and chemotherapy [38,39]. The surgical approach to grade 3 astrocytoma is determined by factors such as the tumor’s location, size, and involvement in critical brain structures [10,38]. The techniques used can vary from traditional surgical approaches, such as craniotomy, to minimally invasive endoscopic methods like the Endoscopic Endonasal Approach (EEA) and the Endoscopic Transorbital Approach (ETOA). The EEA and ETOA are two minimally invasive strategies that may be employed depending on the tumor’s location. The EEA involves accessing the tumor through the nasal passages and sphenoid sinus, offering a direct route to tumors located in the anterior skull base, sellar, and parasellar regions. The ETOA, on the other hand, provides access to tumors located in the orbital, periorbital, and anterior cranial fossa regions. The EEA and ETOA avoid external incisions and reduce tissue damage and recovery time by avoiding larger craniotomies. Both endoscopic approaches require a highly skilled multidisciplinary team, including neurosurgeons, otolaryngologists, and ophthalmologists, to carefully navigate critical structures [40]. In this clinical case, a traditional craniotomy was performed based on the surgeon’s competencies and available technical support. The main diagnostic steps, which begin with a patient presenting with symptoms and involve developing an examination and treatment plan, including operative therapy, followed by specific treatment and further follow-up of the patient, are highlighted in the flowchart (Figure 7). 

## 4. Conclusions

Overall, the specific genetic alterations resulting from RAS mutations in anaplastic astrocytoma can influence a tumor’s behavior, prognosis, and response to targeted therapies. Identifying these alterations through molecular profiling of the tumor tissue can help guide treatment decisions and personalized therapeutic approaches for patients with anaplastic astrocytoma [10]. Future studies will be needed to determine the convergence or involvement of the different RASopathies and intracranial tumors mentioned in Table 1. 

The surgical management of intracranial astrocytoma involves careful planning depending on the tumor’s location. The techniques used can vary from traditional surgical approaches such as craniotomy to minimally invasive endoscopic methods like the ETOA and EEA. The goal is to achieve maximal safe resection to facilitate further adjuvant therapies and improve patient outcomes [40].

## Figures and Tables

**Table 1:** Literature review of Schimmelpenning syndrome’s association with intracranial tumors.

Disorder	Tumor Type	Reference
Schimmelpenning syndrome	Astrocytoma	Chiang MC et al. [2], 2019
Schimmelpenning-like syndrome (unclear)	Astrocytoma	Watanabe et al. [11], 2016
Schimmelpenning syndrome	Parenchymal cyst	Kamate et al. [12], 2009

**Table 2:** Literature review of another similar syndrome’s association with intracranial tumors.

Disorder	Tumor Type	Reference
Epidermal nevus syndrome	Lipoma; Medulloblastoma	De Vito et al. [13] 2021
Organoid nevus syndrome	Scleral choristoma	Shields et al. [14], 2014
Encephalocraniocutaneous lipomatosis	Lipoma	Chandravanshi [15], 2014
Epidermal nevus syndrome	Medulloblastoma	Okumura et al. [16], 2012
Epidermal nevus syndrome	Complex choristoma; Arachnoid cyst, Scleral osteoma	Sharma et al. [3], 2012
Phakomatosis pigmentovascularis-like (Unclear)	Optic glioma	Seifert et al. [17], 2012
Linear sebaceous nevus syndrome	Arachnoid cyst	Nour I. et al. [18], 2012
Linear sebaceous nevus syndrome	Complex choristoma	Lin and Yan [19], 2010
Linear sebaceous nevus syndrome	Complex choristoma	Park et al. [20], 2009
Linear sebaceous nevus syndrome	Choroidal hemangioma	Yan et al. [21], 2007
Epidermal nevus syndrome	Left orbital and cerebellopontine angle cistern lipomas	Canyigit and Oguz [22] 2006
Encephalocraniocutaneous lipomatosis	Lipoma	Mall et al. [23], 2000
Linear sebaceous nevus syndrome	Complex conjunctival choristoma	Brodsky et al. [24], 1997
Linear sebaceous nevus syndrome	Hamartoma of the right lateral ventricle	Barth et al. [25], 1977
Epidermal nevus syndrome	Choroidal osteoma; complex choristoma	Mullaney and Weatherhead [26], 1996
Linear sebaceous nevus syndrome	Cerebellar venous angioma	Seawright et al. [27], 1996
Linear sebaceous nevus syndrome	Optic glioma	Sato et al. [28], 1994
Encephalocraniocutaneous lipomatosis	Lipoma	Grimalt et al. [29], 1993
Organoid nevus syndrome	Meningo-encephalo-angioneurinomatosis	Clancy et al. [30], 1985
Linear sebaceous nevus syndrome	Choroid plexus papilloma; Anterior horn ependyma	Levin et al. [31], 1984
Nevus sebaceous	Hamartoma	Moskowitz and Honig [32], 1982
Nevus sebaceus	Meningoencephaloangioneurinomatosis	Moskowitz R et al. [32] 1982
Epidermal nevus syndrome	Gliomatosis	Choi and Kudo [33], 1981
Linear sebaceous nevus syndrome	Right lateral ventricle hamartoma	Barth et al. [25], 1977
Nevus unius lateralis	Glioma	Andriola [34], 1976
Linear sebaceous nevus syndrome	Leptomeningeal hemangioma	Mollica et al. [35], 1974
Nevus unius lateralis	Astrocytoma	Meyerson [36], 1967

## Data Availability

Data are contained within the article.
